# Assessing the Association of Physician and Specialist Maldistribution with Out-of-hospital Cardiac Arrest Outcomes: Implications for Regulatory Policy

**DOI:** 10.31662/jmaj.2024-0241

**Published:** 2025-02-28

**Authors:** Atsushi Takayama, Hemant Poudyal

**Affiliations:** 1 Department of Pharmacoepidemiology, Graduate School of Medicine and Public Health, Kyoto University, Kyoto, Japan; 2 Population Health and Policy Research Unit, Kyoto University Graduate School of Medicine, Kyoto, Japan

**Keywords:** healthcare disparity, mortality, physician maldistribution

## Abstract

**Introduction:**

Because regional physician maldistribution is considered a potential contributor to disparities in healthcare outcomes, several countries regulate the number of physicians and specialists per region to ameliorate health disparities. However, the association between regional physician maldistribution and specific outcomes, such as out-of-hospital cardiac arrest (OHCA) at the regional level, remains unclear. This study aims to evaluate the association between regional physician and specialist maldistribution and OHCA outcomes.

**Methods:**

This ecological study used 12 years of longitudinal public open datasets in Japan. We examined the disparity trends of indices of physician and specialist (emergency physicians, cardiologists, and cardiac surgeons) distribution using the Gini index. We also examined the physician uneven distribution index, a newly introduced policy index incorporating local demand and supply of medical services. Next, we analyzed the association between these distributions and OHCA-related outcomes (30-day survival rate and 30-day favorable neurological outcome).

**Results:**

The overall number of physicians and each specialist steadily increased throughout all regions and the observation period, but the trends in the regional distribution of specialists for each region were not always synchronized with the distribution of overall physicians. Although the disparity within each index has gradually decreased, the disparity of specialists remained high compared with overall physicians. Moreover, regional physician distributions, which showed the lowest level of disparity across regions, were consistently associated with OHCA-related outcomes, whereas the regional disparity of specialists, which consistently exhibited a higher level of disparity, was not associated with the outcomes.

**Conclusions:**

Paradoxically, the unevenly distributed specialist distribution indices did not reflect their relevant outcomes, despite their direct involvement in the specific outcomes. Therefore, our findings call into question the validity of policies aimed at correcting the total number of physicians without considering the impact of specialists on healthcare outcomes.

## Introduction

Regional disparities in the outcomes of out-of-hospital cardiac arrests (OHCAs) persist globally ^(1)^, despite improved access to high-quality cardiopulmonary resuscitation (CPR), automatic external defibrillators (AEDs), and basic life support. The average incidence of OHCA among adults attending emergency medical services is 96 adults per 100,000 person-years, with a survival rate of 5.6% to discharge, but with regional variations ^(2)^. For example, the highest OHCA incidence is in Australia, followed by North America, Europe, and Asia, whereas the lowest survival to discharge rate is in Asia and the highest is in Australia ^(2)^. A more recent meta-analysis reported similar trends, with a higher survival rate of 8% among patients who received CPR ^(3)^. There is also a noticeable disparity in OHCA outcomes between urban and rural areas, with better outcomes in urban than in rural areas in many countries ^(4), (5)^.

The disparities in outcomes from OHCAs are largely attributed to the local community’s comprehensive healthcare system, given the success of resuscitation efforts hinges on the chain of survival including quality of bystander CPR by professional and lay people, emergency medical services (EMS) response time, prompt defibrillation, advanced life support, and post-resuscitation care ^(6)^. Previous research suggests that the better OHCA outcomes in urban areas may be due to a higher prevalence of individuals trained in CPR, a more robust EMS infrastructure, an efficient transportation network, and elevated standards of post-resuscitation care―all of which can significantly affect OHCA outcomes ^(7)^.

Given the aforementioned regional disparity of OHCA outcomes and the pivotal role played by the local community’s comprehensive system in such outcomes, it is imperative that greater scientific attention is directed toward not merely the urban vs rural dichotomy defined by population density, but rather toward addressing the “maldistribution of healthcare professionals” across regions to rectify the disparity in OHCA outcomes between urban and rural areas. This is because population density is not a readily modifiable factor, and therefore, a more practical index that accurately reflects the imbalanced distribution of medical demand and supply in each region is required to enhance the regional parity of healthcare outcomes between urban and rural areas. However, there is a dearth of literature investigating the relationship between regional disparity of healthcare professionals and unfavorable OHCA outcomes. Prior studies have relied solely on population density to analyze the regional disparity of OHCA outcomes between urban and rural areas ^(7)^.

The Ministry of Health, Labour, and Welfare (MHLW) in Japan implemented the physician uneven distribution index (PUDI) in 2019 to rectify disparities in healthcare outcomes through the regulation of physician allocation across regions ^(8)^. Nevertheless, the association between PUDI, which accounts for local demand and supply of medical services at the tertiary medical area level, and a specific regional healthcare outcome such as OHCA remains uncertain. Thus, it is imperative to determine whether regional physician distribution indices such as PUDI and the number of physicians per 100,000 population (NPPP) accurately reflect healthcare outcomes and thereby establish their usefulness as policy indices at the regional level. Moreover, examining the association between specialist distribution and OHCA outcomes, considering their direct involvement in OHCA patient care, is equally important.

Therefore, the central objective of this study is to examine the association between indices of physician distribution, specifically PUDI and NPPP, and indices of specialist distribution, specifically, the number of emergency physicians per 100,000 population (NEPP), the number of cardiologists per 100,000 population (NCPP), and the number of certified cardiac surgeons per 100,000 population (NCSPP), and the 30-day survival rate and the incidence of favorable neurological outcomes after OHCA, at the tertiary medical area level.

## Materials and Methods

### Study design and data sources

This study uses a longitudinal, ecological design and national census data obtained from the Portal Site of Official Statistics of Japan (as per the supplemental resources). The geographical unit of analysis was established as the tertiary medical area because this unit is the designated focus of policy efforts aimed at correcting disparities in physician distribution.

### Exposure measures

The exposures of interest were the following five indices of physician distribution in the geographical unit: PUDI, NPPP, NEPP, NCPP, and NCSPP. All data were obtained from the National Physician Census conducted by MHLW in 2018. In 2019, MHLW publicly disclosed tertiary medical area data using 2018 data, aiming to use PUDI as a policy index to evaluate physician maldistribution. PUDI incorporates three supplementary variables in comparison with conventional headcount-based indices such as NPPP. The age- and sex-adjusted consultation rate, in addition to population inflow/outflow during working hours, serves as an index of medical demand, whereas the age- and sex-adjusted working time of physicians is used as a gauge of medical supply for each area. Consequently, a low PUDI value suggests a deficit of physician supply relative to medical demand. At the time of this study, only one year of PUDI data was available; thus, exposure variable data were limited to 2018 for statistical analysis. The detailed description of PUDI and its formula have been previously documented elsewhere ^(9)^.

### Outcome measures

We analyzed 30-day survival rates and 30-day favorable neurological outcomes of OHCA occurring between 2008 and 2020. The data were restricted to OHCA events of cardiac origin that were witnessed by bystanders. A favorable neurological outcome was defined as a Cerebral Performance Category of 1 (conscious and alert with optimal cerebral function) or 2 (conscious and alert with moderate but satisfactory cerebral function) and an Overall Performance Category of 1 or 2, assessed 30 days post admission ^(10)^. A bystander was defined as an individual who either visually or auditorily witnessed the cardiac arrest event and the patient’s vital signs before the arrival of EMS.

### Covariates

On the basis of previous studies, we selected the following covariates to include in our analysis: population density ^(11)^, proportion of the population aged 65 years or older, mean annual household income, designated emergency hospital density ^(12)^ as a proxy for the distance between the location of the OHCA event and the nearest hospital, traffic volume ^(13)^, and mean emergency transportation time (EMTT) ^(14)^ in each geographical unit. The proportion of individuals aged 65 or older was derived from the national census ^(15)^. Population density, calculated as the number of people per prefectural area, was obtained from the Geospatial Information Authority of Japan, Ministry of Land, Infrastructure, Transport, and Tourism ^(16)^. The mean annual household income was extracted from the Basic Survey on Wage Structure ^(17)^. The density of designated emergency hospitals was calculated by dividing the number of such facilities in a prefecture, obtained from the Medical Facility Prefectural Survey ^(18)^, by the prefectural area (km^2^) reported by the Ministry of Land, Infrastructure, Transport, and Tourism ^(19)^. Traffic volume, represented as the number of vehicles on designated roads from 7 AM to 7 PM on a weekday divided by road length ^(20)^, was obtained from the road traffic census conducted by the Ministry of Land, Infrastructure, Transport, and Tourism ^(21)^. All data were collected in 2018 except for traffic volume, given the traffic census is conducted every five years, and the most recent available data were collected in 2015. The EMTT was calculated as the elapsed time from the receipt of the emergency call by EMS to arrival at the designated hospital. The data were collected by certified emergency medical paramedics using the Utstein Style ^(22)^ and verified by official prefectural data managers to rectify any inconsistencies. The data were obtained from the annual Current State of Emergency Transport and Rescue Report published by the Fire and Disaster Management Agency. Further information on these variables, including available web links, is provided in the supplementary file.

### Statistical analysis

The baseline characteristics of the tertiary medical area data were stratified into four quartiles (Q1-Q4) of the PUDI index. These characteristics were expressed as mean ± standard deviation (SD). The longitudinal trends of the NPPP, NEPP, NCPP, and NCSPP indices and the disparities measured by the Gini index were graphed from 2010 to 2020, which was the longest available data period. The outcomes were also graphed for each PUDI quartile. The disparities in the outcomes were analyzed longitudinally using the Gini index. The association between the indices of physician and specialist distribution and OHCA outcomes was estimated using multivariable linear regression with Generalized Estimation Equations (GEEs). We used GEE to estimate the coefficients and 95% confidence intervals (CIs) with an identity link function, assumed residuals in a Gaussian distribution, exchanged the working correlation matrix, and used robust standard estimation. As a sensitivity analysis, a cross-sectional analysis was conducted using multiple linear regression for the association between the indices of physician and specialist distribution and OHCA outcomes only in 2018. A two-tailed p < 0.01 was deemed statistically significant after accounting for multiple testing with a Bonferroni correction. The data were analyzed using Stata version 17.1 and were obtained from publicly accessible, anonymized databases. Ethical approval was waived in accordance with the Ministory of Health, Labor and Welfare’s Ethical Guidelines for Medical and Health Research Involving Human Subjects.

## Results

The results of the descriptive statistics are listed in Table 1. The mean and SDs of the PUDI for all 47 areas combined were 230.5 ± 37.7, with a median of 231.0, and interquartile range (IQR) was 204.7 to 254.3. The mean and SD of the NPPP for all areas was 264.3 ± 42.6, with a median of 263.7, and IQR was 232.2 to 300.1. As the PUDI quartiles increased, the proportion of people aged 65 years or older, emergency hospital density, and EMTT decreased, whereas other variables, including OHCA outcomes, increased. The mean ± SD [median (IQR)] of emergency medical transportation time for all cases in 2018 were 37.9 ± 4.1 [38.2 (35.4-39.5)]. Population density in the fourth stratum received a remarkably high score. The mean ± SD [median (IQR)] of the 30-day survival rate and favorable neurological outcome of OHCA in 2018 were 13.4 ± 3.6 [13.3 (11.5-15.6)] and 8.8 ± 2.4 [8.8 (7.5-10)], respectively, whereas the corresponding values for the study period of 2008-2020 were 12.2 ± 3.6 [11.8 (9.8-14.2)] and 7.9 ± 2.7 [7.6 (5.9-9.3)].

**Table 1.** Characteristics of Tertiary Medical Areas Stratified by Quartile of PUDI, Mean (SD).

Variables	PUDI - Q1	PUDI - Q2	PUDI - Q3	PUDI - Q4	Total
Interquartile range	(169.3-177.4)	(177.4-231.0)	(231.0-279.3)	(279.3-329)	
	N = 12	N = 12	N = 12	N = 11	N = 47
**PUDI**	184.4 (12.4)	218.8 (8.7)	242.4 (7.2)	280.4 (24.4)	230.5 (37.7)
**NPPP (per 100,000 people)**	216.5 (20.1)	254.5 (18.9)	276.4 (30.1)	314.0 (27.3)	264.3 (42.6)
**NEPP (per 100,000 people)**	2.22 (0.65)	2.58 (1.20)	2.97 (0.86)	3.26 (0.83)	2.75 (0.99)
**NCPP (per 100,000 people)**	8.01 (1.28)	10.20 (1.47)	11.16 (2.03)	11.75 (1.86)	10.25 (2.20)
**NCSPP (per 100,000 people)**	2.08 (0.29)	2.52 (0.37)	2.49 (0.47)	3.00 (0.52)	2.51 (0.53)
**Proportion of people aged ≥65 years (%)**	30.9 (2.6)	30.4 (2.3)	30.0 (2.9)	28.8 (3.6)	30.1 (2.9)
**Population density (1,000 people/km^2^)**	0.44 (0.55)	0.35 (0.36)	0.61 (0.97)	1.35 (2.07)	0.67 (1.22)
**Annual household Income (¥ × 10,000)**	286 (24)	290 (20)	293 (24)	298 (35)	292 (27)
**Density of designated emergency hospitals (km^2^/hospital)**	173.8 (114.8)	115.2 (72.8)	98.1 (66.2)	76.3 (50.7)	116.7 (87.8)
**Traffic volume (×1,000 cars/12 hours)**	6.12 (2.62)	5.54 (2.01)	6.36 (3.25)	7.76 (4.73)	6.42 (3.37)
**The mean EMTT for all emergency transportation cases (min)**	40.6 (3.9)	36.6 (3.3)	38.0 (2.6)	36.4 (5.2)	37.9 (4.1)
**The 30-day survival rate of OHCA (%)**	11.5 (3.0)	13.0 (3.0)	13.0 (3.1)	16.2 (4.2)	13.4 (3.6)
**The 30-day favorable neurological outcome of OHCA in 2018 (%)**	7.8 (2.3)	8.3 (2.1)	8.8 (2.2)	10.6 (2.6)	8.8 (2.4)
**The mean of 30-day survival rate of OHCA from 2008 to 2020, (%)**	10.3 (2.3)	11.6 (3.1)	12.8 (3.2)	14.4 (4.4)	12.2 (3.6)
**The mean 30-day favorable neurological outcome of OHCA from 2008 to 2020, (%)**	6.7 (2.0)	7.5 (2.3)	8.3 (2.8)	9.2 (3.2)	7.9 (2.7)

EMTT: emergency transportation time, FNO: favorable neurological outcome, NEPP: number of emergency physicians per 100,000 population, NCPP: number of cardiologists per 100,000 population, NCSPP: number of cardiac surgeons per 100,000 population, NPPP: number of physicians per 100,000 people, OHCA: out-of-hospital cardiac arrest, PUDI: physician uneven distribution index, Q: quartile, SD, standard deviation.

**Figure 1.**
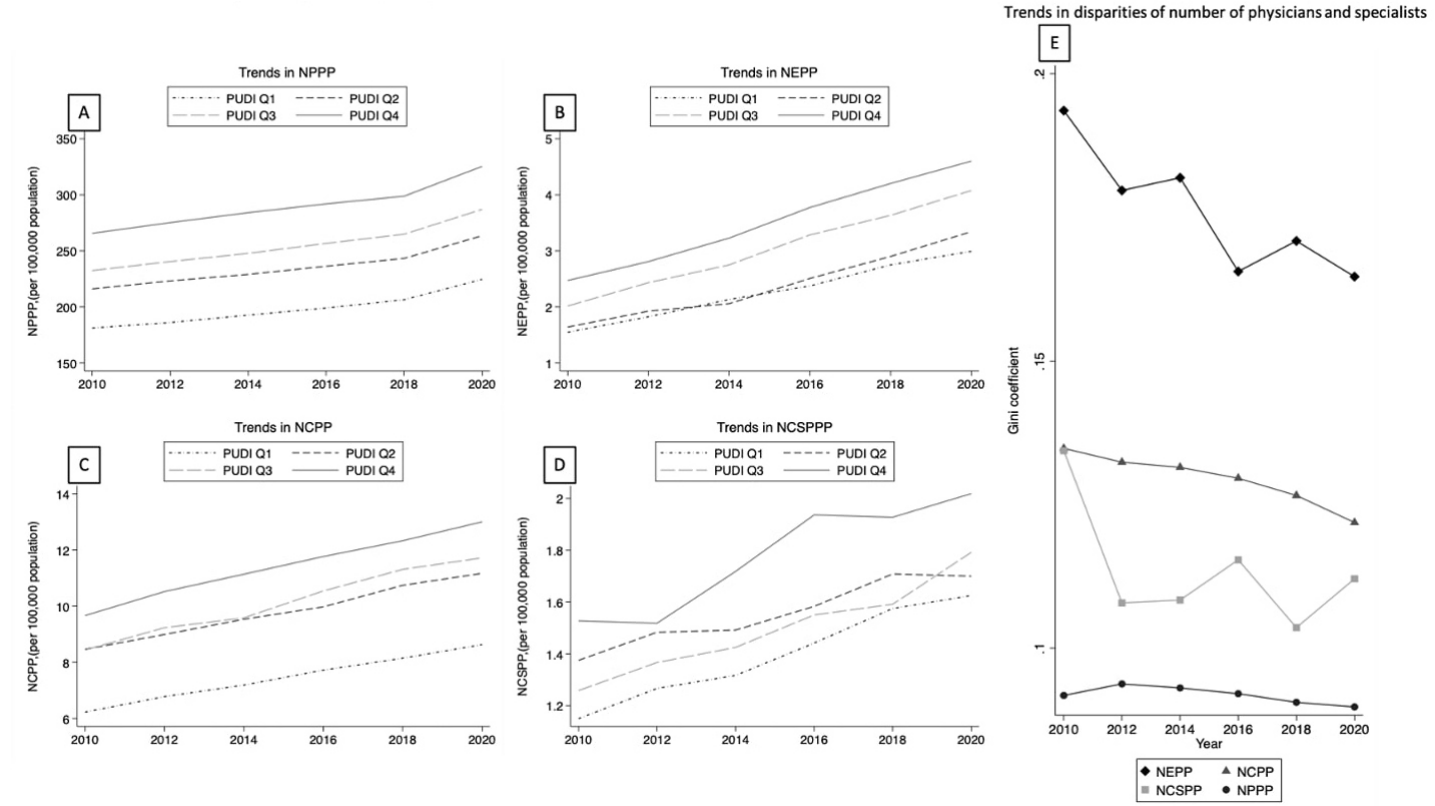
Trends in regional distribution index of (A) NPPP, (B) NEPP, (C) NCPP, and (D) NCSPP stratified by Physician Uneven distribution Index quartiles; and (E) trends in Gini Index of each index from 2010 to 2020. NCPP: number of cardiologists per 100,000 population, NCSPP: number of cardiac surgeons per 100,000 population, NEPP: number of emergency physicians per 100,000 population, NPPP: number of physicians per 100,000 population, Q: quartile.

The longitudinal trends in the regional distribution indices of overall physicians and specialists by PUDI quartiles, in addition to each index’s Gini index, are depicted in Figure 1. The trend of NPPP was nearly identical across all PUDI quartiles over a 10-year period. However, the regional distribution of specialists was not uniform among the PUDI quartiles. Although the disparity within each index has decreased gradually, the disparity of specialists remained high compared with that of overall physicians.

**Figure 2.**
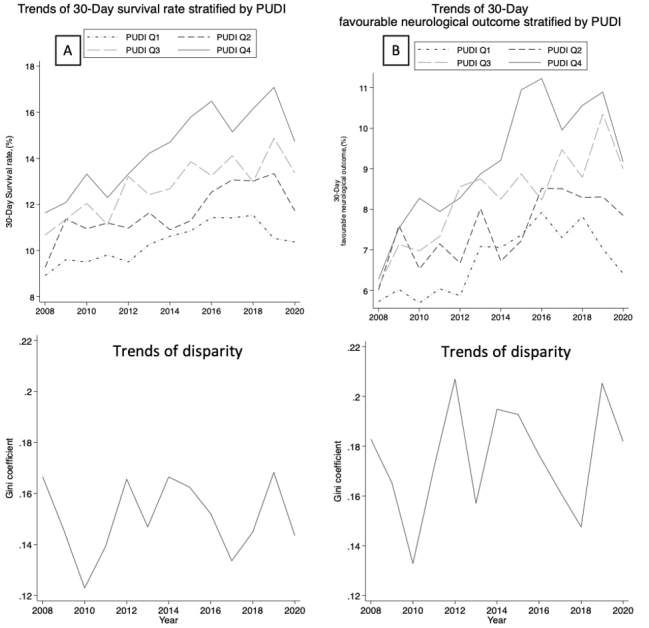
Trends in (A) 30-day survival rate of out-of-hospital cardiac arrest and (B) 30-day favorable neurological outcome of out-of-hospital cardiac arrest stratified by PUDI quartiles EMTT: emergency transportation time, OHCA: out-of-hospital cardiac arrest, PUDI: physician uneven distribution index, Q: quartile.

Figure 2 displays the 12-year longitudinal trends of the 30-day survival rate and favorable neurological outcome of OHCA by PUDI quartiles, along with the Gini index of each index. The 30-day survival rate and favorable neurological outcome showed gradual improvement, but the trends were similar among all PUDI quartiles, with no obvious trend of improvement in terms of outcome disparity.

The association between regional physician distribution indices, the 30-day survival rate, and favorable neurological outcome of OHCA over 12 years (2008-2020) was analyzed. Results of the adjusted multivariable analysis indicated that PUDI (adjusted coefficient: 0.043, 95% CI: 0.022-0.065, p < 0.001), NPPP (adjusted coefficient: 0.036, 95% CI: 0.018-0.054, p < 0.001), and NCSPP (adjusted coefficient: 1.438, 95% CI: 0.180-2.695, p = 0.025) were positively and independently associated with the 30-day survival rate of OHCA (Table 2). However, NEPP (adjusted coefficient: 0.531, 95% CI: −0.112 to 1.173, p = 0.105) and NCPP (adjusted coefficient: 0.273, 95% CI: −0.024 to 0.569, p = 0.071) were not associated with the 30-day survival rate.

**Table 2.** The Association between Regional Physician/Specialist Distribution Index and 30-Day Survival Rate of Out-of-Hospital Cardiac Arrest over 12 Years Using GEE.

Indices of physician and specialists’ distribution	Crude coefficient	95% CIs		*p* Values	Adjusted coefficient	95% CIs		*p* Values
**PUDI**	**0.038**	**0.021**	**0.056**	**<0.001**	**0.043**	**0.022**	**0.065**	**<0.001**
Proportion of people aged ≥65 years, (%)	-	-	-	-	−0.112	−0.447	0.223	0.512
Population density (1,000 people/km**^2^**)	-	-	-	-	−1.757	−3.255	−0.259	0.021
Annual household Income (¥ × 10,000)	-	-	-	-	0.000	−0.035	0.035	0.996
Density of designated emergency hospitals (Km^2/hospital)	-	-	-	-	0.011	0.002	0.019	0.018
Traffic volume (×1,000 cars/12 hours)	-	-	-	-	0.723	0.083	1.362	0.027
EMTT (min)	-	-	-	-	−0.229	−0.388	−0.069	0.005
**NPPP**	**0.021**	**0.004**	**0.038**	**0.017**	**0.036**	**0.018**	**0.054**	**<0.001**
Proportion of people aged ≥65 years, (%)	-	-	-	-	−0.406	−0.710	−0.103	0.009
Population density (1,000 people/km**^2^**)	-	-	-	-	−1.751	−3.253	−0.249	0.022
Annual household Income (¥ × 10,000)	-	-	-	-	0.005	−0.030	0.040	0.777
Density of designated emergency hospitals (Km^2/hospital)	-	-	-	-	0.011	0.002	0.020	0.012
Traffic volume (×1,000 cars/12 hours)	-	-	-	-	0.711	0.072	1.350	0.029
EMTT (min)	-	-	-	-	−0.253	−0.407	−0.099	0.001
**NEPP**	**0.859**	**0.162**	**1.556**	**0.016**	**0.531**	**−0.112**	**1.173**	**0.105**
Proportion of people aged ≥65 years, (%)	-	-	-	-	−0.275	−0.648	0.098	0.148
Population density (1,000 people/km**^2^**)	-	-	-	-	−0.229	−1.619	1.160	0.746
Annual household Income (¥ × 10,000)	-	-	-	-	0.005	−0.035	0.045	0.824
Density of designated emergency hospitals (Km^2/hospital)	-	-	-	-	0.005	−0.004	0.014	0.309
Traffic volume (×1,000 cars/12 hours)	-	-	-	-	0.127	−0.491	0.745	0.687
EMTT (min)	-	-	-	-	−0.374	−0.530	−0.217	< 0.001
**NCPP**	**0.283**	**−0.057**	**0.624**	**0.103**	**0.273**	**−0.024**	**0.569**	**0.071**
Proportion of people aged ≥65 years, (%)	-	-	-	-	−0.429	−0.769	−0.089	0.013
Population density (1,000 people/km**^2^**)	-	-	-	-	−0.192	−1.533	1.149	0.779
Annual household Income (¥ × 10,000)	-	-	-	-	−0.002	−0.041	0.037	0.915
Density of designated emergency hospitals (Km^2/hospital)	-	-	-	-	0.005	−0.004	0.014	0.278
Traffic volume (×1,000 cars/12 hours)	-	-	-	-	0.102	−0.493	0.696	0.737
EMTT (min)	-	-	-	-	−0.346	−0.507	−0.184	< 0.001
**NCSPP**	**1.847**	**0.488**	**3.206**	**0.008**	**1.438**	**0.180**	**2.695**	**0.025**
Proportion of people aged ≥65 years, (%)	-	-	-	-	−0.318	−0.659	0.022	0.067
Population density (1,000 people/km**^2^**)	-	-	-	-	−0.574	−2.002	0.853	0.431
Annual household Income (¥ × 10,000)	-	-	-	-	0.005	−0.034	0.044	0.789
Density of designated emergency hospitals (Km^2/hospital)	-	-	-	-	0.005	−0.004	0.014	0.253
Traffic volume (×1,000 cars/12 hours)	-	-	-	-	0.225	−0.387	0.838	0.471
EMTT (min)	-	-	-	-	−0.331	−0.491	−0.170	<0.001

**Notation.** Adjusted for proportion of people aged 65 years or older, population density, mean annual household income, density of designated emergency hospital, traffic volume, EMTT. The *p*-value cutoff after Bonferroni correction is 0.05/5 hypotheses (=0.01). **PUDI**: physician uneven distribution index, **NPPP**: number of physicians per 100,000 people, **NEPP**: number of emergency physicians per 100,000 population, **NCPP**: number of cardiologists per 100,000 population,** NCSPP**: number of cardiac surgeons per 100,000 population, CI: confidence interval, EMTT: emergency transportation time (represents the length of time between receiving the emergency call by EMS and arriving at the selected hospital), GEE: generalized estimation equation.

In the examination of the association between regional physician distribution indices and the 30-day favorable neurological outcome of OHCA over the observational period, only PUDI (adjusted coefficient: 0.025, 95% CI: 0.009-0.041, p = 0.003) and NPPP (adjusted coefficient: 0.022, 95% CI: 0.008-0.035, p = 0.002) were independently associated with the 30-day favorable neurological outcome (Table 3). Meanwhile, indices of regional specialist distribution (NEPP, NCPP, and NCSPP) were not associated with the 30-day favorable neurological outcome (Table 3).

**Table 3.** The Association between Regional Physician/Specialist Distribution Index and 30-Day Favorable Neurological Outcome of Out-of-Hospital Cardiac Arrest over 12 Years Using GEE.

Indices of physician and specialists’ distribution	Crude coefficient	95% CIs		*p* Values	Adjusted coefficient	95%CIs		*p* Values
**PUDI**	**0.024**	**0.012**	**0.036**	**<0.001**	**0.025**	**0.009**	**0.041**	**0.003**
Proportion of people aged ≥65 years, (%)	-	-	-	-	−0.060	−0.312	0.192	0.641
Population density (1,000 people/km^2)	-	-	-	-	−0.890	−2.017	0.237	0.122
Annual household Income (¥×10,000)	-	-	-	-	−0.006	−0.032	0.020	0.663
Density of designated emergency hospitals (Km^2/hospital)	-	-	-	-	0.005	−0.002	0.011	0.174
Traffic volume (×1,000 cars/12 hours)	-	-	-	-	0.401	−0.081	0.882	0.103
EMTT (min)	-	-	-	-	−0.167	−0.287	−0.047	0.006
**NPPP**	**0.014**	**0.002**	**0.026**	**0.016**	**0.022**	**0.008**	**0.035**	**0.002**
Proportion of people aged ≥ 65 years, (%)	-	-	-	-	−0.227	−0.454	−0.001	0.049
Population density (1,000 people/km**^2^**)	-	-	-	-	−0.938	−2.058	0.181	0.1
Annual household Income (¥ × 10,000)	-	-	-	-	−0.003	−0.029	0.024	0.838
Density of designated emergency hospitals (Km^2/hospital)	-	-	-	-	0.005	−0.001	0.012	0.121
Traffic volume (×1,000 cars/12 hours)	-	-	-	-	0.414	−0.062	0.890	0.088
EMTT (min)	-	-	-	-	−0.177	−0.292	−0.062	0.003
**NEPP**	**0.572**	**0.093**	**1.052**	**0.019**	**0.347**	**−0.111**	**0.806**	**0.137**
Proportion of people aged ≥65 years, (%)	-	-	-	-	−0.142	−0.408	0.124	0.296
Population density (1,000 people/km**^2^**)	-	-	-	-	−0.065	−1.057	0.927	0.897
Annual household Income (¥ × 10,000)	-	-	-	-	−0.003	−0.031	0.026	0.853
Density of designated emergency hospitals (Km^2/hospital)	-	-	-	-	0.001	−0.005	0.008	0.675
Traffic volume (×1,000 cars/12 hours)	-	-	-	-	0.080	−0.361	0.521	0.721
EMTT (min)	-	-	-	-	−0.248	−0.359	−0.136	<0.001
**NCPP**	**0.149**	**−0.087**	**0.385**	**0.216**	**0.098**	**−0.118**	**0.314**	**0.374**
Proportion of people aged ≥65 years, (%)	-	-	-	-	−0.234	−0.482	0.014	0.064
Population density (1,000 people/km**^2^**)	-	-	-	-	0.096	−0.881	1.073	0.847
Annual household Income (¥ × 10,000)	-	-	-	-	−0.007	−0.035	0.022	0.636
Density of designated emergency hospitals (Km^2/hospital)	-	-	-	-	0.001	−0.006	0.008	0.776
Traffic volume (×1,000 cars/12 hours)	-	-	-	-	0.010	−0.424	0.443	0.965
EMTT (min)	-	-	-	-	−0.243	−0.361	−0.125	<0.001
**NCSPP**	**1.007**	**0.050**	**1.964**	**0.039**	**0.542**	**−0.385**	**1.470**	**0.252**
Proportion of people aged ≥65 years, (%)	-	-	-	-	−0.193	−0.444	0.058	0.132
Population density (1,000 people/km**^2^**)	-	-	-	-	−0.056	−1.109	0.996	0.916
Annual household Income (¥ × 10,000)	-	-	-	-	−0.004	−0.033	0.025	0.78
Density of designated emergency hospitals (Km^2/hospital)	-	-	-	-	0.001	−0.005	0.008	0.752
Traffic volume (×1,000 cars/12 hours)	-	-	-	-	0.059	−0.392	0.511	0.796
EMTT (min)	-	-	-	-	−0.237	−0.355	−0.119	<0.001

**Notation.** Adjusted for proportion of people aged 65 years or older, population density, mean annual household income, density of designated emergency hospital, traffic volume, and EMTT. The p-value cutoff after Bonferroni correction is 0.05/5 hypotheses (=0.01). **PUDI**: physician uneven distribution index, **NPPP**: number of physicians per 100,000 people, **NEPP**: number of emergency physicians per 100,000 population, **NCPP**: number of cardiologists per 100,000 population,** NCSPP**: number of cardiac surgeons per 100,000 population, CI: confidence interval, EMTT: emergency transportation time (represents the length of time between receiving the emergency call by EMS and arriving at the selected hospital), GEE: generalized estimation equation.

The sensitivity analysis affirmed the robustness of the association between the overall physician distribution index and OHCA outcomes, and the absence of association between the specialist distribution index and such outcomes (Supplementary Tables S1 and S2).

## Discussion

### Statement of principal findings

Over the past decade, there has been a steady increase in the number of physicians and specialists involved in providing care to patients with OHCA in Japan. Moreover, disparities within each distribution index have generally improved, yet the disparity across indices persists. Interestingly, only regional distribution indices exhibiting low disparities were found to be associated with OHCA-related outcomes, whereas indices of specialists with high disparities were not, despite their direct role in the provision of care to patients with OHCA in real-world settings. Furthermore, although the 30-day survival rate and 30-day favorable neurological outcome have shown improvement, as reported in prior studies ^(1)^, the disparity of these outcomes has not substantially decreased over the past 12 years across all tertiary medical areas.

### Interpretation within the context of the wider literature

The apparent paradox that although the overall physician distribution indices consistently reflect the OHCA-related outcomes, specialist distribution indices do not, warrants cautious interpretation. This discrepancy, however, may shed light on the complex dynamics between regional physician distribution and OHCA outcomes. A similar paradox has been reported in previous literature, in which researchers found no clear association between avoidable mortality and overall physician supply in a country-and town-level ecological study involving data from 19 OECD countries ^(23), (24)^. One plausible interpretation is that overall physician distribution indices act as proxies for regional healthcare system accessibility or minor components of a broader framework, rather than as direct determinants of healthcare outcomes. When physician distribution indices primarily reflect healthcare accessibility across regions, but their granularity is insufficient to capture healthcare access within specific geographic units, these indices may fail to exhibit a clear association with particular outcomes despite their underlying relevance. In other words, physician distribution indices may fail to reflect healthcare outcomes, particularly in areas where such outcomes matter most. The availability of an integrated system, comprising highly trained personnel, medical equipment, and organizational collaboration, is widely recognized as a critical factor influencing OHCA outcomes ^(25)^. In contrast, the number of physicians and specialists represents only one facet of this comprehensive system. Our observational study does not aim to establish a causal relationship between regional physician maldistribution and OHCA outcomes or vice versa, but it highlights that uneven distribution indices may not accurately reflect relevant healthcare outcomes in some cases. Therefore, relying too heavily on a single aspect of the healthcare system without accounting for the broader picture increases the risk of policy intervention failure. Healthcare policies, especially those influenced by political motivations, must be grounded in scientific evidence ^(26)^ and designed with clear, goal-oriented objectives ^(27)^.

### Implications for policy, practice, and research

Furthermore, MHLW is contemplating expanding the utilization of a PUDI-style approach to rectifying specialist distribution per region to ameliorate the significant inequity in access to specialized medical care across tertiary medical areas ^(8)^. Considering these findings, the discrepancy in the association between regional distribution and specialized healthcare outcomes between the overall number of physicians and specialists is significant in the context of political efforts to address regional imbalances in medical supply. Ironically, the implementation of PUDI methods for regional specialist distribution may not be effective unless there is an equitable distribution of specialists. Nonetheless, this is evidently not the case. Consequently, it seems premature to adjust the number of specialists per region without considering specific healthcare outcomes or the impact of multiple other factors relevant to a specific outcome.

Considering the well-established association between the number of specialists and OHCA outcomes at the individual level in previous research ^(28)^, our findings may evoke a suspicion regarding ecological fallacy. However, the present study aimed to scrutinize the relationship between regional distribution indices and specific healthcare outcomes in the geographic unit as a political target, with the goal of evaluating the validity of regulations that use regional physician distribution indices to improve disparities in healthcare outcomes. Thus, whether the number of specialists affects OHCA outcomes at the individual level is beyond the scope of our inquiry. If the physician distribution indices never reflect healthcare outcomes even predictively, merely adjusting only the number of physicians in each region will not produce improved outcomes. Moreover, disregarding the comprehensive nature of the healthcare system can lead to ill-advised political interventions and cause unwarranted upheaval.

### Strengths and limitations

The present study has several strengths. Firstly, given the pivotal role of bystanders in OHCA outcomes ^(29)^, this study has concentrated its focus on the outcomes of OHCA in the presence of a bystander. This focus controls the variability in pre-hospital conditions, such as the prevalence of bystander CPR training or AED accessibility between regions and minimizes related bias. Secondly, the analysis is restricted to cardiogenic OHCA, reducing the impact of intricate factors other than the EMS and eliminating non-modifiable etiologies of OHCA, which can have a broad range of variabilities depending on each case ^(30)^. In addition, the study uses validated long-term, repeated-outcome data from multiple independent national agencies, such as the MHLW, Fire and Disaster Management Agency, and Ministry of Land, Infrastructure, Transport and Tourism, increasing the statistical significance of the results despite the limited number of tertiary medical areas.

However, the study also has several limitations. Firstly, our analysis does not include some well-established factors that influence OHCA outcomes, such as the presence of an emergency physician at the site ^(31)^, the accessibility of AEDs ^(32)^, the prevalence of bystander CPR training ^(33)^, the quality of post-resuscitation care ^(34)^, socioeconomic status ^(35)^, and population health measures ^(36)^. Secondly, our results cannot be interpreted as representing associations at the individual level because this ecological study investigates the association of regional physician distribution indices on OHCA outcomes at the regional level, with the aim of validating regulatory policies in tertiary medical areas. Thirdly, although the main analysis incorporates repeated outcome variables using GEE to improve statistical power, it has a cross-sectional nature, given some outcome variables were recorded either before or concurrently with the exposure variables. Considering these limitations, our observational study cannot be interpreted establishing a causal relationship. Thus, it would be valuable to investigate the effects of interventions targeting the distribution of healthcare professionals on OHCA outcomes through a prospective controlled study design, such as a cluster-randomized pragmatic trial, in a future study ^(37)^. Lastly, because we only used data from Japan, our findings cannot be generalized to other countries with different healthcare systems, organizational structures, and resource allocation challenges.

In conclusion, the current policy indices of specialist distribution did not appropriately reflect the specific healthcare outcomes that these indices should reflect. Therefore, rectifying the number of specialists per region according to those indices may be baseless. Healthcare policies aimed at regulating physician numbers must recognize the comprehensiveness of the healthcare system and regional disparities and should use indices meticulously crafted to optimize specific healthcare outcomes.

## Article Information

### Conflicts of Interest

None

### Author Contributions

Atsushi Takayama: conceptualization, data curation, writing―original draft, methods, formal analysis

Hemant Poudyal: conceptualization, funding acquisition, supervision, writing―review and editing, methods

## Supplement

Supplementary Materials
