# Supplementary material for: Assessing the Association of Physician and Specialist Maldistribution with Out-of-hospital Cardiac Arrest Outcomes: Implications for Regulatory Policy
Source: JMA J. 2025 Feb 28;8(2):506–16. doi: 10.31662/jmaj.2024-0241 (PMC12095126; doi:10.31662/jmaj.2024-0241)
Supplement: Supplementary Materials [file 2433-3298-8-2-0506-s001.pdf]

**Supplementary Materials**

*Assessing the Association of Physician and Specialist Maldistribution with Out-of-Hospital Cardiac Arrest Outcomes: Implications for Regulatory Policy*

Atsushi TAKAYAMA, Hemant POUDYAL

**Contents:**

- **Table S1.** The association between regional physician/specialist distribution index and 30-day survival rate of out-of-hospital cardiac arrest in 2018
- **Table S2.** The association between regional physician/specialist distribution index and 30-day favorable neurological outcome of out-of-hospital cardiac arrest in 2018
- **Data resources**

**Table S1.** The association between regional physician/specialist distribution index and 30-day survival rate of out-of-hospital cardiac arrest in 2018

| | Crude Coef. | 95%CIs | | *p* values | Adjusted Coef. | 95%CIs | | *p* values |
|---|---|---|---|---|---|---|---|---|
| **PUDI** | **0.042** | **0.016** | **0.068** | **0.002** | **0.053** | **0.014** | **0.091** | **0.008** |
| Proportion of people aged ≥ 65 years, | – | – | – | – | 0.092 | -0.507 | 0.691 | 0.757 |
| Population density (1,000 | – | – | – | – | -2.919 | -5.597 | -0.240 | 0.034 |
| Annual household Income (¥×10,000) | – | – | – | – | 0.036 | -0.026 | 0.099 | 0.248 |
| Density of designated emergency | – | – | – | – | 0.012 | -0.004 | 0.027 | 0.136 |
| Traffic volume (×1,000 cars / 12 hours) | – | – | – | – | 1.104 | -0.041 | 2.248 | 0.058 |
| EMTT (min) | – | – | – | – | -0.192 | -0.477 | 0.094 | 0.182 |
| **NPPP** | **0.022** | **-0.003** | **0.046** | **0.083** | **0.046** | **0.014** | **0.078** | **0.006** |
| Proportion of people aged ≥ 65 years, | – | – | – | – | -0.265 | -0.803 | 0.272 | 0.324 |
| Population density (1,000 | – | – | – | – | -3.024 | -5.683 | -0.364 | 0.027 |
| Annual household Income (¥×10,000) | – | – | – | – | 0.043 | -0.019 | 0.105 | 0.172 |
| Density of designated emergency | – | – | – | – | 0.013 | -0.003 | 0.029 | 0.098 |
| Traffic volume (× 1,000 cars / 12 hours) | – | – | – | – | 1.134 | 0.003 | 2.265 | 0.049 |
| EMTT (min) | – | – | – | – | -0.213 | -0.487 | 0.060 | 0.123 |
| **NEPP** | **1.052** | **0.072** | **2.033** | **0.036** | **0.927** | **-0.150** | **2.004** | **0.090** |
| Proportion of people aged ≥ 65 years, | – | – | – | – | -0.039 | -0.664 | 0.586 | 0.900 |
| Population density (1,000 | – | – | – | – | -1.332 | -3.662 | 0.999 | 0.255 |
| Annual household Income (¥×10,000) | – | – | – | – | 0.045 | -0.022 | 0.112 | 0.182 |
| Density of designated emergency | – | – | – | – | 0.006 | -0.010 | 0.021 | 0.461 |
| Traffic volume (×1,000 cars / 12 hours) | – | – | – | – | 0.492 | -0.543 | 1.528 | 0.342 |
| EMTT (min) | – | – | – | – | -0.357 | -0.620 | -0.095 | 0.009 |
| **NCPP** | **0.489** | **0.055** | **0.924** | **0.028** | **0.549** | **-0.085** | **1.183** | **0.088** |
| Proportion of people aged ≥ 65 years, | – | – | – | – | -0.350 | -0.930 | 0.231 | 0.231 |
| Population density (1,000 | – | – | – | – | -1.505 | -3.925 | 0.915 | 0.216 |
| Annual household Income (¥×10,000) | – | – | – | – | 0.014 | -0.056 | 0.084 | 0.685 |
| Density of designated emergency | – | – | – | – | 0.009 | -0.008 | 0.025 | 0.285 |
| Traffic volume (×1,000 cars / 12 hours) | – | – | – | – | 0.618 | -0.483 | 1.719 | 0.263 |
| EMTT (min) | – | – | – | – | -0.210 | -0.543 | 0.122 | 0.208 |
| **NCSPP** | **2.143** | **-1.235** | **5.521** | **0.208** | **1.241** | **-2.355** | **4.837** | **0.489** |
| Proportion of people aged ≥ 65 years, | – | – | – | – | -0.191 | -0.811 | 0.429 | 0.537 |
| Population density (1,000 | – | – | – | – | -0.834 | -3.257 | 1.589 | 0.491 |
| Annual household Income (¥×10,000) | – | – | – | – | 0.038 | -0.031 | 0.107 | 0.273 |
| Density of designated emergency | – | – | – | – | 0.003 | -0.012 | 0.018 | 0.689 |
| Traffic volume (×1,000 cars / 12 hours) | – | – | – | – | 0.274 | -0.789 | 1.336 | 0.605 |
| EMTT (min) | – | – | – | – | -0.364 | -0.642 | -0.085 | 0.012 |

**Notation.** Adjusted for proportion of people aged 65 years or older, population density, mean annual household income, density of designated emergency hospital, traffic volume, EMTT. CI: confidence interval, PUDI: physician uneven distribution index, NPPP: number of physicians per 100,000 people, EMTT: mean emergency travel time (It represents the length of time between receiving the emergency call by EMS and arriving at the selected hospital.) NEPP: number of certified emergency physician Per 100,000 population, NCPP: number of cardiologists per 100,000 population, NCSPP: number of cardiac surgeons per 100,000 population, The *p*-value cut-off after Bonferroni correction is 0.05 / 5 hypotheses (= 0.01).

**Table S2.** The association between regional physician/specialist distribution index and 30-day favorable neurological outcome of out-of-hospital cardiac arrest in 2018

| | Crude Coef. | 95%CIs | | *p* values | Adjusted Coef. | 95%CIs | | *p* values |
|---|---|---|---|---|---|---|---|---|
| **PUDI** | **0.028** | **0.011** | **0.046** | **0.002** | **0.031** | **0.005** | **0.057** | **0.021** |
| Proportion of people aged ≥ 65 years, (%) | – | – | – | – | -0.060 | -0.464 | 0.344 | 0.765 |
| Population density (1,000 people/km^2) | – | – | – | – | -1.777 | -3.584 | 0.030 | 0.054 |
| Annual household Income (¥×10,000) | – | – | – | – | 0.018 | -0.025 | 0.060 | 0.407 |
| Density of designated emergency | – | – | – | – | 0.004 | -0.007 | 0.014 | 0.452 |
| Traffic volume (×1,000 cars / 12 hours) | – | – | – | – | 0.600 | -0.171 | 1.372 | 0.124 |
| EMTT (min) | – | – | – | – | -0.110 | -0.302 | 0.083 | 0.256 |
| **NPPP** | **0.013** | **-0.003** | **0.030** | **0.118** | **0.028** | **0.007** | **0.050** | **0.011** |
| Proportion of people aged ≥ 65 years, (%) | – | – | – | – | -0.270 | -0.630 | 0.090 | 0.137 |
| Population density (1,000 people/km^2) | – | – | – | – | -1.919 | -3.700 | -0.137 | 0.035 |
| Annual household Income (¥×10,000) | – | – | – | – | 0.022 | -0.020 | 0.063 | 0.301 |
| Density of designated emergency | – | – | – | – | 0.005 | -0.005 | 0.016 | 0.329 |
| Traffic volume (× 1,000 cars / 12 hours) | – | – | – | – | 0.649 | -0.108 | 1.407 | 0.091 |
| EMTT (min) | – | – | – | – | -0.117 | -0.300 | 0.066 | 0.204 |
| **NEPP** | **0.640** | **-0.027** | **1.307** | **0.060** | **0.414** | **-0.313** | **1.141** | **0.256** |
| Proportion of people aged ≥ 65 years, (%) | – | – | – | – | -0.168 | -0.589 | 0.254 | 0.426 |
| Population density (1,000 people/km^2) | – | – | – | – | -0.724 | -2.296 | 0.848 | 0.358 |
| Annual household Income (¥×10,000) | – | – | – | – | 0.021 | -0.024 | 0.067 | 0.349 |
| Density of designated emergency | – | – | – | – | 0.000 | -0.010 | 0.010 | 0.986 |
| Traffic volume (×1,000 cars / 12 hours) | – | – | – | – | 0.191 | -0.508 | 0.889 | 0.584 |
| EMTT (min) | – | – | – | – | -0.212 | -0.388 | -0.035 | 0.02 |
| **NCPP** | **0.286** | **-0.011** | **0.583** | **0.059** | **0.286** | **-0.139** | **0.711** | **0.181** |
| Proportion of people aged ≥ 65 years, (%) | – | – | – | – | -0.313 | -0.703 | 0.076 | 0.112 |
| Population density (1,000 people/km^2) | – | – | – | – | -0.879 | -2.502 | 0.744 | 0.28 |
| Annual household Income (¥×10,000) | – | – | – | – | 0.006 | -0.041 | 0.053 | 0.802 |
| Density of designated emergency | – | – | – | – | 0.002 | -0.009 | 0.013 | 0.74 |
| Traffic volume (×1,000 cars / 12 hours) | – | – | – | – | 0.283 | -0.455 | 1.022 | 0.443 |
| EMTT (min) | – | – | – | – | -0.132 | -0.355 | 0.090 | 0.237 |
| **NCSPP** | **1.875** | **-0.371** | **4.121** | **0.100** | **1.317** | **-1.035** | **3.670** | **0.264** |
| Proportion of people aged ≥ 65 years, (%) | – | – | – | – | -0.194 | -0.600 | 0.211 | 0.339 |
| Population density (1,000 people/km^2) | – | – | – | – | -0.733 | -2.318 | 0.853 | 0.356 |
| Annual household Income (¥×10,000) | – | – | – | – | 0.020 | -0.025 | 0.065 | 0.375 |
| Density of designated emergency | – | – | – | – | -0.001 | -0.011 | 0.009 | 0.864 |
| Traffic volume (×1,000 cars / 12 hours) | – | – | – | – | 0.182 | -0.513 | 0.878 | 0.599 |
| EMTT (min) | – | – | – | – | -0.198 | -0.380 | -0.016 | 0.034 |

**Notation.** Adjusted for proportion of people aged 65 years or older, population density, mean annual household income, density of designated emergency hospital, traffic volume, EMTT. CI: confidence interval, PUDI: physician uneven distribution index, NPPP: number of physicians per 100,000 people, EMTT: mean emergency travel time (It represents the length of time between receiving the emergency call by EMS and arriving at the selected hospital.) NEPP: number of certified emergency physician Per 100,000 population, NCPP: number of cardiologists per 100,000 population, NCSPP: number of cardiac surgeons per 100,000 population, The p-value cut-off after Bonferroni correction is 0.05 / 5 hypotheses (= 0.01).

**Data resources**

All data in this research are available from the following links.

Exposure variables:

Tertiary medical area (prefectural level) data for NPPP, NEPP, NCMPP, and NCSPP were extracted from the National Physician Census conducted by MHLW in 2018.

- National Physician Census conducted by MHLW at 2018
    - Ministry of Health, Labour and Welfare. Statistics of doctors, dentists and pharmacists. 2018. (https://www.e-stat.go.jp/stat-search/files?page=1&layout=datalist&toukei=00450026&tstat=000001135683&cycle=7&year=20180&tclass1=000001135684&tclass2=000001135686&tclass3val=0).
- Physician uneven distribution index (PUDI)
    - Ministry of Health, Labour and Welfare. 医師偏在指標 [Uneven distribution of physicians]. 2020. Accessed 12 Dec 2021. https://www.mhlw.go.jp/content/10801000/000480270.pdf

Outcome variables:

Data for the 30-day survival rate and the 30-day favorable neurological outcome of OHCA from 2008 to 2020 was obtained from the Current State of Emergency Transport and Rescue Report, annually published by Fire and Disaster Management Agency (FDMA).

- The Current State of Emergency Transport and Rescue Report, annually published by Fire and Disaster Management Agency (FDMA)
    - Fire and Disaster Management Agency, Ministry of Internal Affairs and Communications. Fire service whitepaper. n.d. Accessed 06 June 2022. https://www.fdma.go.jp/publication/#whitepaper

Covariates:

The proportion of the≥65 years old population at the tertiary medical area level was derived from the national census.

- The proportion of the≥65 years old population at 2018

- Statistics Bureau, Ministry of Internal Affairs and Communications. Population estimates (as of October 1, 2018) by age and gender. 2019. Accessed 06 June 2022. https://www.stat.go.jp/data/jinsui/2018np/index.html

Population density, defined by the number of individuals divided by each area, was obtained from the Geospatial Information Authority of Japan, Ministry of Land, Infrastructure, Transport, and Tourism (MLIT).

- Geospatial Information Authority of Japan, Ministry of Land, Infrastructure, Transport, and Tourism (MLIT)
  - Statistics Bureau, Ministry of Internal Affairs and Communications. Statistical Handbook of Japan 2021. 2021. Accessed 06 June 2022. https://www.stat.go.jp/english/data/handbook/c0117.html

Mean annual household income was extracted from the Basic Survey on Wage Structure.

- Basic Survey on Wage Structure at 2018
  - Ministry of Health, Labour and Welfare. Basic statistical survey on wage structure. 2018. Accessed 13 Aug 2021. https://www.e-stat.go.jp/stat-search/files?page=1&layout=datalist&toukei=00450091&tstat=000001011429&cycle=0&year=20180&tclass1=000001113395&tclass2=000001113397&tclass3=000001113406&tclass4val=0

The density of designated emergency hospitals was calculated using the number of designated emergency hospitals in the area available from the Medical Facility Prefectural Survey divided by the tertiary medical area(km2) as reported by MLIT.

Medical Facility Prefectural Survey at 2018

- Ministry of Health, Labour and Welfare. Medical Facility Prefectural Survey. 2018. Accessed 06 June 2022. https://www.e-stat.go.jp/stat-search/file-download?statInfId=000031862115&fileKind=1

Traffic volume, defined by the number of cars on the designated road from 7 AM to 7 PM on a weekday, divided by the length of the designated road, was retrieved from the road traffic census performed by MLIT.

- Definition of Traffic volume
  - Ministry of Land, Infrastructure, Transport and Tourism. General traffic volume survey. 2015. Accessed 06 June 2022. https://www.mlit.go.jp/road/census/h27/data/pdf/kasyorep.pdf

Traffic census is a quinquennial nationwide survey, and the nearest available data of 2018 were collected during 2015.

- Road traffic census at 2015
  - Ministry of Land, Infrastructure, Transport and Tourism. National Road and Street Traffic Conditions Survey General Traffic Volume Survey Summary Table of Tabulation Results. 2015. Accessed 06 June 2022. https://www.mlit.go.jp/road/census/h27/data/xlsx/syuukei04.xlsx
